# Association of Malaysian-MIND Diet Scores with Brain Activation in Older Adults: A Functional MRI Study

**DOI:** 10.3390/biomedicines14061238

**Published:** 2026-05-29

**Authors:** Muhamad Mustaqim M Zapawi, Yee Xing You, Mazlyfarina Mohamad, Ponnusamy Subramaniam, Mohd Razif Shahril, Faizah Mohd Zaki, Suzana Shahar

**Affiliations:** 1 Dietetic Program, Centre for Healthy Ageing and Wellness (H-CARE), Faculty of Health Sciences, Universiti Kebangsaan Malaysia, Jalan Raja Muda Abdul Aziz, Kuala Lumpur 50300, Malaysia; p131177@siswa.ukm.edu.my (M.M.M.Z.); suzana.shahar@ukm.edu.my (S.S.); 2 Diagnostic Imaging and Radiotherapy Program, Faculty of Health Sciences, Universiti Kebangsaan Malaysia, Jalan Raja Muda Abdul Aziz, Kuala Lumpur 50300, Malaysia; mazlyfarina@ukm.edu.my; 3 Clinical Psychology and Behavioural Health Program, Centre for Healthy Ageing and Wellness (H-CARE), Faculty of Health Sciences, Universiti Kebangsaan Malaysia, Jalan Raja Muda Abdul Aziz, Kuala Lumpur 50300, Malaysia; ponnusaami@ukm.edu.my; 4 Nutrition Program, Centre for Healthy Ageing and Wellness (H-CARE), Faculty of Health Sciences, Universiti Kebangsaan Malaysia, Jalan Raja Muda Abdul Aziz, Kuala Lumpur 50300, Malaysia; razifshahril@ukm.edu.my; 5 Department of Radiology, Tunku Ampuan Besar Tuanku Aishah Rohani Hospital, Universiti Kebangsaan Malaysia, Jalan Yaacob Latif, Kuala Lumpur 56000, Malaysia; drfaizah@ppukm.ukm.edu.my

**Keywords:** Malaysian-MIND diet, DLPFC, brain activation, fMRI, older adult

## Abstract

**Background/Objectives**: Cognitive flexibility and working memory are regulated by the dorsolateral prefrontal cortex (DLPFC), which is closely linked to the progression of cognitive decline. The Mediterranean-DASH Intervention for the Neurodegenerative Delay (MIND) diet shows potential to lower cognitive decline risk in older adults. This study aimed to examine the association between Malaysian-MIND diet (MY-MINDD©) scores with brain activation among Malaysian older adults. **Methods**: A cross-sectional study was conducted among forty older adults aged 60–75 years. Subjects were stratified into quartiles of MY-MINDD© scores with ten subjects per quartile. Dietary intake was evaluated utilising a validated 124-item semiquantitative Food Frequency Questionnaire (FFQ). Brain activation was measured using task-based fMRI (N-back and Stroop Colour Word Test). DLPFC activation was analysed in Brodmann’s areas 9, 46, and the anterior cingulate cortex (ACC). ANCOVA and multiple linear regression were used to evaluate brain activation differences across MY-MINDD© quartiles, accommodating for gender, age, education, and body mass index (BMI). **Results**: Subjects in the highest MY-MINDD© quartile had significantly greater DLPFC activation during 0-back, 1-back, and SCWT incongruent tasks (*p* < 0.05). Higher MY-MINDD© adherence is linked to better task performance (*p* < 0.001). Multivariate General Linear Model (GLM) revealed a significant overall effect on brain activation (Pillai’s Trace = 0.544, F(8,27) = 4.11, *p* = 0.003). Multiple linear regression demonstrated significant positive associations between MY-MINDD© scores and DLPFC activation (*p* < 0.0125). **Conclusions**: Higher adherence to the MY-MINDD© diet was associated with greater brain activation, suggesting its relevance as a proxy for identifying risk of cognitive decline.

## 1. Introduction

Cognitive decline represents a major global health challenge, particularly in ageing populations [1]. With the ageing population, the incidence of dementia is predicted to increase from 55 million to 75 million by 2030, and 131 million by 2050 [2]. In Malaysia, the rate of Mild Cognitive Impairment (MCI) as well as dementia among older adults aged 60 years and above is recorded at 8.5% and 16.0%, respectively [3]. As the brain ages, structural changes are commonly recognised as markers of distinguishing normal ageing from dementia, where healthy ageing is typically accompanied by gradual declines in episodic memory, processing speed, and executive function [4].

The DLPFC is a crucial brain area responsible for executive functions, including working memory, attention, planning, and processing speed, which are among the earliest domains affected in age-related cognitive decline and MCI [5,6]. It is present in the middle frontal gyrus of humans (i.e., the lateral region of Brodmann’s area, i.e., BA 9 and BA 46. BA 9 consisted of the inferior and superior frontal gyrus, while BA46 consisted of the middle frontal gyrus [7]. Due to its high metabolic demand, the DLPFC is particularly vulnerable to oxidative stress and neuroinflammatory processes associated with ageing [8]. Impaired synaptic neuroplasticity caused by a dysfunctional DLPFC could lead to MCI [9]. Nutrition is essential for maintaining brain health [10]. Studies showed that a well-rounded diet abundant in nutrients such as omega-3 fatty acids, polyphenols, and certain vitamins supports neuronal integrity and synaptic plasticity [9,11]. Evidence from functional magnetic resonance imaging (fMRI) studies using tasks such as N-back and SCWT indicates that healthier dietary intake is associated with enhanced working memory function and more efficient activation of prefrontal regions, including the DLPFC. In contrast, excess intake of certain fats, sugars, and sodium can impair it [12,13,14].

Diet has been acknowledged as a modifiable risk factor for neurodegeneration [10]. The MIND diet has attracted attention, given its link to slower cognitive decline as well as a decreased risk of Alzheimer’s disease (AD) [15]. However, most evidence is derived from Western populations, limiting generalizability across diverse cultural and dietary contexts in Southeast Asian countries such as Malaysia [16]. To address this gap, the Malaysian adaptation of the MIND diet, MY-MINDD©, was developed by incorporating culturally relevant foods, such as tropical fruits, local green leafy vegetables (e.g., *ulam*), legumes, and soy products [17]. MY-MINDD© emphasises the consumption of antioxidant- and anti-inflammatory-rich foods, which may mitigate the neurodegenerative process and support the functional integrity of prefrontal networks [17]. Emerging studies have demonstrated promising links between adherence to the MIND diet as well as cognitive outcomes, such as the Digit Span, Mini-Mental State Examination (MMSE), Rey Auditory Verbal Learning Test (RAVLT), Digit Symbol, and Visual Reproduction; demonstrating its potential for risk assessment for cognitive decline [15,17,18]. However, the above-mentioned neuropsychological batteries are subjective measures that may be influenced by factors such as educational background, cultural bias, and motivation during testing [19]. Additionally, they provide only indirect assessment of cognitive function and may not fully capture underlying neural mechanisms or subtle changes in the early stages of cognitive decline [20]. Therefore, examining DLPFC activation provides a biologically plausible neural correlate to investigate the potential mechanisms linking MY-MINDD© adherence to cognitive function.

fMRI is an objective measure that offers a distinctive opportunity to identify subtle diet-related changes in brain activity during specific cognitive tasks, even prior to the onset of clinical symptoms of cognitive impairment [21]. Previous fMRI studies have demonstrated that adherence to brain-healthy foods such as *ulam* consumption (a type of traditional salad typically eaten raw by Asian populations, including those in Southeast Asia, Japan, Korea, and India) and other dietary patterns is associated with greater activation in regions supporting executive function and working memory, involving areas such as the prefrontal cortex and parietal lobes [9,14,22,23]. In addition, emerging neuroimaging evidence suggests that dietary patterns rich in antioxidants, polyphenols, and anti-inflammatory nutrients are associated with enhanced neural efficiency and functional activation during cognitively demanding tasks. However, no study to date has explored the connection between adherence to the MIND diet and functional brain activity using fMRI. Given that the MIND diet emphasises several neuroprotective food components, examining its association with brain activation may provide important mechanistic insights into diet-related cognitive resilience. Neuroimaging evidence for culturally adapted diets, such as MY-MINDD©, is currently lacking.

A previous study showed that the newly developed MY-MINDD© has a strong correlation with cognitive performance, with higher MY-MINDD© scores associated with better cognitive outcomes, including MMSE, Digit Span, and Visual Reproduction [17]. However, there is a need to further investigate the association between MY-MINDD© and objective measures, such as fMRI, to serve as a proxy for cognitive outcomes in populations other than those used for its development. Therefore, this study aims to examine the association between MY-MINDD© scores and brain activation among a sample of older adults. As far as we are aware, this is among the first studies to investigate the relationship between culturally adapted MIND diet scores and brain activation patterns using fMRI in an Asian older adult population.

## 2. Materials and Methods

### 2.1. Study Design and Sampling

The present cross-sectional study included 40 subjects (aged 60–75 years) who had previously undergone eligibility screening according to the inclusion and exclusion criteria. A stratified sampling method was used, whereby subjects were categorised into quartiles of MY-MINDD© scores, representing increasing levels of adherence to the MY-MINDD©, where quartile 1 (Q1) is the lowest adherence and quartile 4 (Q4) is the highest adherence, with ten subjects selected from each quartile to ensure balanced representation across the distribution. Quartile cut-off points were determined based on the distribution of MY-MINDD© scores within the study sample, based on a prior validation study [17]. This approach was used to facilitate comparison across different levels of MY-MINDD© adherence. The inclusion criteria for this study were adults aged 60 years and above who were proficient in Malay or English. Meanwhile, the exclusion criteria included individuals with a history of major neurological or psychiatric disorders (such as schizophrenia, AD, or stroke), significant physical disabilities that could interfere with study procedures, or contraindications to MRI, including claustrophobia, the presence of electronic implants, or metallic foreign bodies. Medical history and basic health profiles were self-reported based on subjects’ recent visit to a medical facility. Subjects were screened prior to enrolment to ensure eligibility according to the study criteria. The sample size was determined using the formula [24]:

$$n={\left[\frac{Z\alpha +Z\beta }{C}\right]}^{2}+3$$

where Zα = 1.96 (corresponding to a 95% confidence level), Zβ = 0.842 (corresponding to 80% statistical power), *r* = 0.42 (as reported in [25], and C = 0.5 × ln[(1 + r)/(1 − r)] = 0.45. Hence,

$$n={\left[\frac{\left(1.96+0.842\right)}{0.45}\right]}^{2}+3$$

n=40subjects.

**Figure 1:**
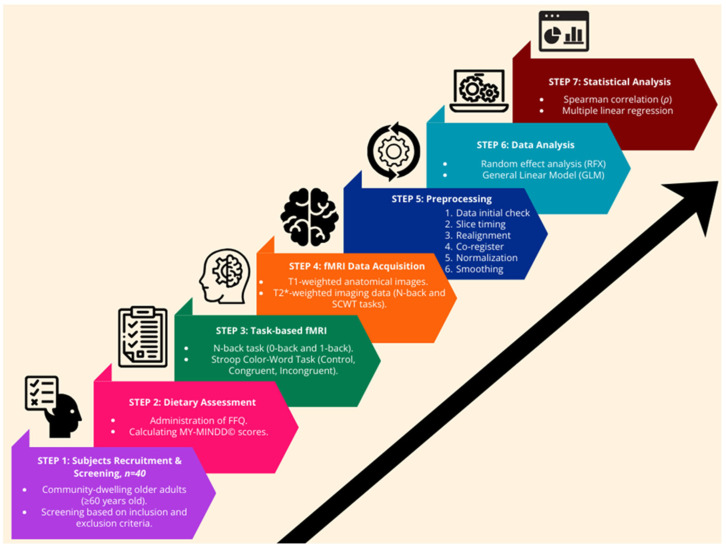
Study’s workflow diagram.

The fMRI scan was performed by a qualified MRI radiographer at an accredited medical imaging facility, with ethical approval (JEP-2023-266) granted by the Medical Research and Ethics Committee. An MRI safety screening checklist was administered and verified by the radiographer prior to scanning to ensure compliance with standard MRI safety protocols. A summary of the study design and workflow diagram is presented in Figure 1.

### 2.2. Adaptation and Validation of MY-MINDD© Scores

MY-MINDD© scores were developed in a previous study as a culturally adapted version of the original MIND diet to better reflect local dietary practices, food availability, and sociocultural context in Malaysia [17]. The adaptation process involved modifying food components to incorporate commonly consumed Malaysian foods while preserving the core principles of the MIND diet, which emphasised plant-based foods, antioxidants, and limited intake of unhealthy food groups. Specifically, berries were replaced with flavonoid-rich fruits that are more commonly available and affordable in Malaysia, given the limited accessibility of berries in the local context. Olive oil was excluded due to its relatively high cost and low usage among the Malaysian population. Similarly, wine and cheese were excluded, as they are infrequently consumed in this population due to cultural and dietary preferences. In addition, legumes and soy products substituted for beans and nuts to better align with typical consumption patterns among Malaysian older adults. For the discouraged food components, sweets, and fried/fast foods were expanded to include desserts, sweetened traditional *kuih*, beverages, and fried/fast foods, reflecting commonly consumed high-sugar and high-fat foods in Malaysia. MY-MINDD© scores have been previously validated against multiple parameters using a pooled data analysis from a total of 810 subjects. The parameters derived include sociodemographic, health, and lifestyle factors, anthropometric measures, dietary intake, depressive symptoms, functional status, and cognitive function, which were employed for validation against MCI [17].

### 2.3. Procedures

This study was conducted in accordance with the 1964 Declaration of Helsinki and its subsequent amendments. Prior to obtaining informed consent, subjects were given a detailed information sheet outlining the study’s objectives, procedures, instructions for completing the required task, and risks. Aside from using the questionnaires to obtain the sociodemographic background and anthropometric parameters such as BMI (derived from measured weight and height), the trained fieldworkers acquired the subjects’ dietary intake details, which include the type of food consumed, portion sizes, and the frequency of intake, ranging from daily, weekly, to monthly, utilising a validated, 124-item semiquantitative Food Frequency Questionnaire (FFQ). This FFQ was adapted from a previous study [26] and further modified to better suit food components relevant to the MY-MINDD© which consists of 11 food groups, including green leafy vegetables, whole grains, flavonoid-rich fruits, other vegetables, legumes and soy products, fish (not fried), poultry (not fried), sweetened *kuih*, desserts, beverages, butter/margarine, red meat, and fast/fried foods [17]. To assess the intake, each food item was converted into standard serving sizes using the Nutritionist Pro^TM^ (Axxya Systems, Stafford, TX USA) software version 4.0, the Atlas of Food Exchange and Portion Sizes UKM [27], and the Malaysian Food Composition database [28]. The dietary intake data obtained from the FFQ were used to derive MY-MINDD© scores, which reflect subjects’ level of adherence to the MY-MINDD© dietary pattern. Following completion of dietary assessments, task-based functional MRI (fMRI) was performed for each subject to assess brain activation during working memory and cognitive flexibility tasks.

### 2.4. fMRI Protocol

**Figure 2:**
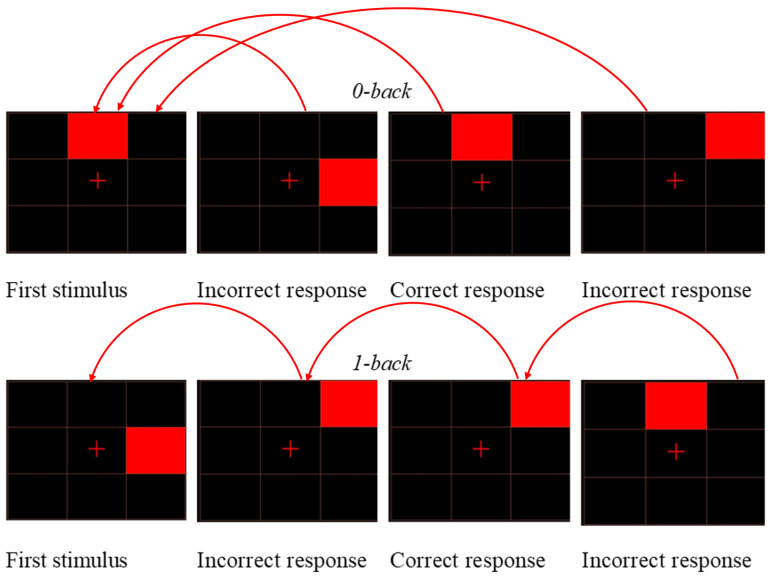
N-back paradigm. Arrows indicate the matching target locations used to determine correct responses during each task condition. In the 0-back condition, subjects identified whether the target location matched a first stimulus; in the 1-back condition, they identified whether the current target location matched the one presented immediately before it. The “+” symbol represents the fixation cross displayed during the inter-block resting period.

The N-back task was employed to evaluate working memory. The two N-back task conditions employed in this study were the 0-back and 1-back conditions used in a prior study [14], which was designed and presented using the Superlab 5 (Cedrus, San Pedro, CA, USA) version 5.0.5. Each of the 0-back and 1-back tasks has four blocks. Each block took 30 s, with a 30-s break in between, making 510 s in total to complete the tasks. Before the fMRI scan, a trained fieldworker would guide the subjects on how to perform the N-back task, which involves responding to a given stimulus and determining whether the target location under 0-back condition was the same as the first block (predefined stimulus) and whether the target location under 1-back condition was the same as the one presented immediately before it, by first displaying to them the four corresponding diagram concerning the 0-back and 1-back blocks of each condition (Figure 2). Correct responses were recorded when subjects accurately identified matching or non-matching target locations according to the task instructions. The reported left and right DLPFC activation values represent brain activation measured during the respective fMRI task conditions, reflecting neural activation associated with working memory and cognitive flexibility processes. Before beginning the 9-min N-back tasks, a trained fMRI radiographer conducted a 6-min structural brain scan.

**Figure 3:**
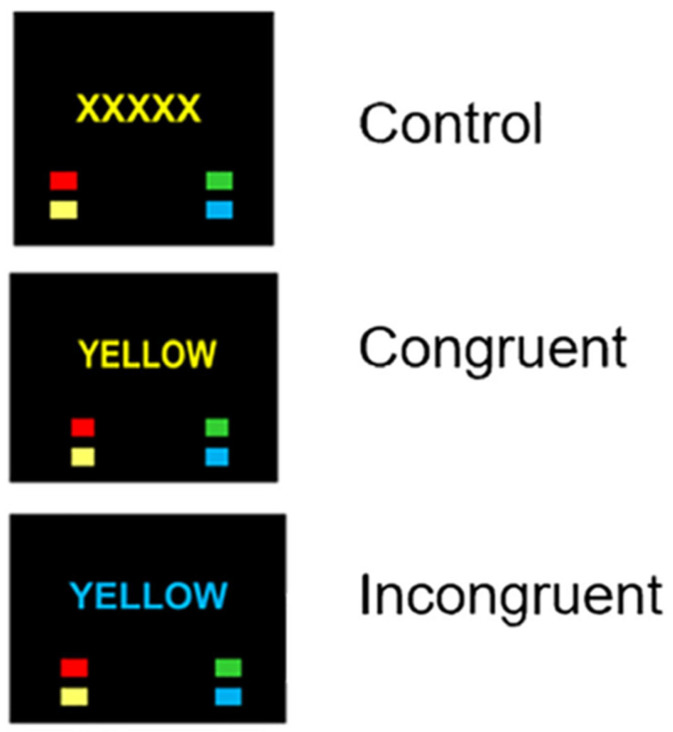
SCWT paradigm. Subjects identified the correct responses according to the font colour of the presented stimuli rather than the word meaning. In the control condition, subjects responded to coloured symbols without semantic interference. In the congruent condition, the font colour matched the word meaning, whereas in the incongruent condition, the font colour differed from the word meaning. The coloured blocks indicate the stimulus colour to which participants were required to respond.

The Stroop Colour Word Test (SCWT) was administered to evaluate cognitive flexibility, including executive function and processing speed. This task included three conditions: control, congruent, and incongruent. Each of these conditions had three task blocks, each with ten trials, as recommended by prior studies [9,29]. Each block lasted 30 s, followed by a 30-s break in between. This task requires 510 s to complete in total. For the SCWT, subjects were shown a series of coloured symbols and words presented through the E-prime system (Psychology Software Tools, Sharpsburg, Pittsburgh, PA, USA) version 3.0 and instructed to respond based on the colour of the font rather than the word’s meaning (Figure 3). In the first paradigm (SCWT-Control), the subjects were first shown a series of coloured ‘X’s (e.g., “XXXXX”) that were displayed in various font colours, and subjects were asked to identify the colour of the ‘X’ symbol. In the second paradigm (SCWT-Congruent), a series of coloured words (e.g., “RED”, “BLUE”, “GREEN”) appeared in matching ink colours (e.g., the word “RED” displayed in red ink), and subjects had to respond to the colour shown. Lastly, in the third paradigm (SCWT-Incongruent), coloured words were shown in a mismatched ink colour (e.g., the word “BLUE” displayed in red ink), requiring subjects to suppress the automatic tendency to read the word and instead indicate the actual font colour. Each stimulus was presented for 3000 ms, and the whole task took around 6 min.

### 2.5. fMRI Data Acquisition

The fMRI data was used to acquire single-shot spin-echo echoplanar imaging (EPI), and the fMRI images were performed on a 3.0T magnetic resonance (MR) scanner (Magnetom Skyra, Siemens, Enlangen, Germany) with each subject being examined for high-resolution T_1_-weighted anatomical images (repetition time [TR] = 1900 ms, echo time [TE] = 2.27 ms, 176 slices, slice thickness = 1 mm) whereas (TR = 3000 ms, TE = 30 ms, flip angle (α) = 90°, 29 slices, slice thickness = 4 mm) for the N-back task and (TR = 2000 ms, TE = 30 ms, flip angle (α) = 90°, 29 slices, slice thickness = 4 mm) for the SCWT task that were performed using T_2_*-weighted imaging data, where T_2_* refers to the effective transverse relaxation that is sensitive to blood oxygen level-dependent (BOLD) signal changes.

### 2.6. Task Performance Data

The percentage of correct response (CR) and the mean response time (RT) on the N-back and SCWT task of each subject were then recorded. The data were captured using the Cedrus Viewer software (Cedrus, CA, USA). Correct response (CR) is the percentage of correct responses from the total responses performed by each subject. The formula of CR is shown as follows:

$$\mathrm{Percentage}\;\mathrm{of}\;\mathrm{correct}\;\mathrm{response}\;(\mathrm{CR})\;=\;\frac{Number\;of\;correct\;responses}{Total\;number\;of\;responses}\times 100\%$$

### 2.7. Preprocessing and Functional Imaging Data Analysis

The expert engineer conducted regular quality control assessments of the MRI machine to ensure the functional images were of high quality. Initial data quality checks were performed to ensure consistency of imaging parameters across subjects and to inspect the image quality, coverage, and orientation of both functional and anatomical images. The Statistical Parametric Mapping (SPM) version 12 (Functional Imaging Laboratory, Wellcome Department of Imaging Neuroscience, Institute of Neurology, University College London, London, UK) software, which was implemented in MATLAB R2023b version 23.2 (MathWorks Inc., Natick, MA, USA), was used for the data preprocessing and analysis procedures. Functional images were initially slice-time corrected and realigned using the mean image of the series, and head motion was assessed using the realignment parameters. Subjects exceeding 1.5 mm translation or 1.5° rotation were flagged for inspection. However, no subject exceeded this threshold. To increase the signal-to-noise ratio by removing high-frequency noise and reducing intersubject variability, these functional images were co-registered to the subject’s mean T1-weighted image and estimated in a standardised Montreal Neurological Institute (MNI) stereotaxic space [30]. After the spatial normalisation process, all of the functional volumes were then subjected to spatial smoothing with a 6 mm full-width at half-maximum isotropic Gaussian kernel. Additional nuisance regression (e.g., white matter/CSF signals) and advanced denoising procedures (e.g., CompCor, ICA-AROMA) were not applied, consistent with typical task-based SPM pipelines.

### 2.8. Group Results Analysis

A group brain activation analysis using a one-sample *t*-test (*p* < 0.05, family-wise error [FWE]-corrected) was carried out using random-effect analysis (RFX). At the first level, individual subject data were fitted using a GLM, where the task conditions for N-back (0-back and 1-back) and SCWT (control, congruent, incongruent) were used as explanatory variables. Motion parameters were included as nuisance covariates, and contrast images representing task-related activation were generated for each subject. These contrast images were subsequently entered into second-level (group) analyses to evaluate group-level brain activation patterns and to examine the association between MY-MINDD© scores and brain activation.

### 2.9. Region of Interest (ROI) Analysis

The WFU PickAtlas classified the DLPFC (Brodmann’s areas 9 and 46) and the ACC, since previous studies had identified this ROI as responsible for the human brain’s working memory and cognitive flexibility [9,22]. To ensure an ROI-based approach, a mask derived from the Automated Anatomical Labelling (AAL) atlas for these regions was applied during activation value extraction, and no whole-brain statistical analysis was performed. After performing individual-subject analysis and a significant correction (*p* < 0.05, familywise error [FWE]-corrected), the ROI brain activation outcome is represented as the respective subjects’ percent signal change.

### 2.10. Statistical Analysis

The IBM SPSS Statistics version 28.0 (IBM Corp., Armonk, NY, USA) software was used for all statistical analyses, with a significance level of *p* < 0.05. Aside from using Shapiro–Wilk to test the data normality (*p* > 0.05), all subjects’ demographic data were presented as frequencies and percentages for categorical variables. Meanwhile, means and standard deviations for continuous variables. To determine significant differences that exist between continuous variables as well as each quartile of the diet score, a one-way ANOVA and Kruskal–Wallis’s test were employed. Meanwhile, for categorical variables, Fisher’s Exact test was applied because of the small expected cell counts. To identify significant differences in the DLPFC activation among the four quartiles of MY-MINDD© scores, a one-way analysis of covariance (ANCOVA) test was applied, controlling for age, gender, years of formal education, and BMI. Spearman’s rank correlations were used to examine the relationships between MY-MINDD© scores, task performance, and brain activation. These correlations were conducted as exploratory analyses to identify candidate fMRI task conditions associated with MY-MINDD© scores. Since these analyses served as preliminary screening steps, no multiple-comparison correction was applied at this stage. Only variables with significant correlations were subsequently included in the multiple linear regression models, where the Bonferroni correction was applied to control for multiple comparisons. A multivariate GLM was performed to assess the overall effect of MY-MINDD© scores on brain activation across multiple fMRI tasks. Multiple linear regression analyses were conducted separately for each fMRI task to examine the association between brain activation and MY-MINDD© scores, while controlling for potential confounders such as gender, age, years of formal education, and BMI. β denotes the correlation value from the analysis. Note that the significance of these multiple linear regressions was evaluated using a Bonferroni-adjusted alpha level to correct for the inflated FWE rates resulting from performing multiple analyses on the same data. The family-wise alpha level (α = 0.05) was categorised by the total number of dependent variables to account for adjustment, using the following formula:

$$P_{\mathrm{corrected}}=\frac{0.05}{4}=0.0125$$

## 3. Results

As depicted in Table 1, the baseline characteristics of the 40 subjects were categorised into quartiles according to the diet score. The average MY-MINDD© score regarding all subjects was 5.68 ± 2.21 (out of a total of 11 scores), with a lower score noted in subjects in the quartile 1 (2.70 ± 0.35), compared to the quartile 4 (8.50 ± 0.71) (*p* < 0.001). Among the sociodemographic and health variables investigated, only BMI was significant. The mean of BMI was higher at the quartile 4 of the MY-MINDD©, compared to the lowest (*p* < 0.05).

Table 2 shows an univariate ANCOVA analysis with the gender, age, years of formal education and BMI adjustments, it also revealed the MY-MINDD© scores at the quartile 3 and quartile 4 demonstrated a notably higher left DLPFC activation for the 0-back (*p* < 0.05, f = 0.25), SCWT incongruent (*p* < 0.01, f = 0.31) tasks and right DLPFC activation with regard to the 0-back (*p* < 0.05, f = 0.22), 1-back (*p* < 0.05, f = 0.22) and the SCWT incongruent (*p* < 0.01, f = 0.36) tasks as compared with the quartile 1 and quartile 2. The left and right DLPFC activation values presented represent task-related brain activation measured using fMRI during the respective cognitive task conditions.

Table 3 shows the relationship between the MY-MINDD© scores and task performance. Significant correlations were observed between the MY-MINDD© scores and correct response percentage for 0-back (*ρ* = 0.536, *p* < 0.001), SCWT control (*ρ* = 0.737, *p* < 0.001), and SCWT congruent (*ρ* = 0.616, *p* < 0.001).

Apart from demonstrating a relationship between MY-MINDD© scores and task performance, Table 4 further illustrates the relationship between task performance and brain activation. Significant correlations were observed between percentage of correct response and DLPFC activation in several tasks: 0-back left (*ρ* = 0.523, *p* = 0.001), 0-back right (*ρ* = 0.425, *p* = 0.006), SCWT control left (*ρ* = 0.326, *p* = 0.040), and SCWT control right (*ρ* = 0.404, *p* = 0.010). Additionally, a significant correlation was observed between response time and left DLPFC activation in the 0-back task (*ρ* = −0.335, *p* = 0.035).

A multivariate GLM demonstrated a substantial overall effect of MY-MINDD© scores on brain activation across multiple fMRI tasks (Pillai’s Trace = 0.544, F(8,27) = 4.11, *p* = 0.003) after adjusting for gender, age, years of formal education, and BMI, as shown in Table 5. Meanwhile, analysis from the multiple linear regression also demonstrated the association between MY-MINDD© scores as being specifically significant with left DLPFC activation while performing 0-back (adjusted R^2^ = 0.28, β = 0.461, *p* = 0.005), SCWT incongruent (adjusted R^2^ = 0.33, β = 0.567, *p* = 0.001) tasks and right DLPFC activation during 0-back (adjusted R^2^ = 0.30, β = 0.564, *p* = 0.001), 1-back (adjusted R^2^ = 0.33, β = 0.480, *p* = 0.003), and SCWT incongruent (adjusted R^2^ = 0.44, β = 0.654, *p* < 0.001) after adjusted for the gender, age, years of formal education and BMI (Table 5).

**Figure 4:**
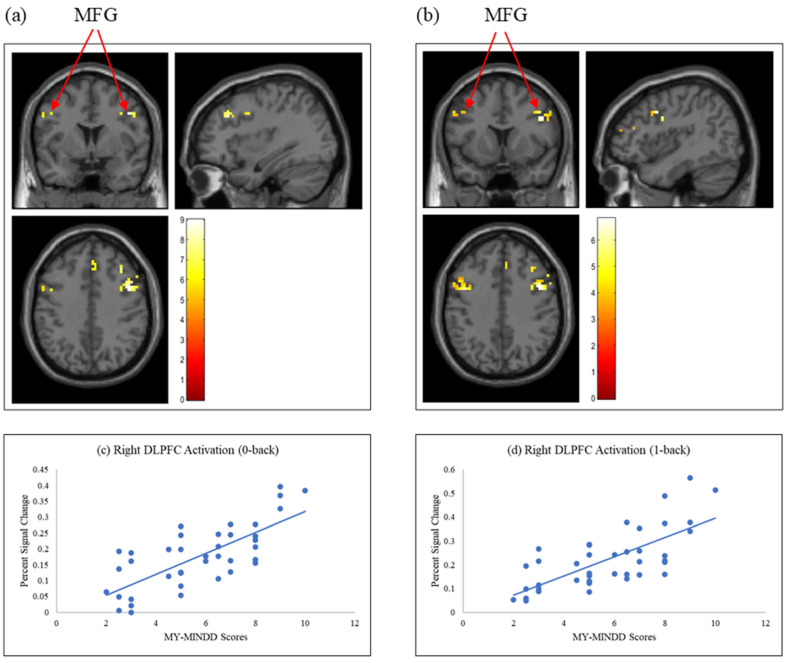
Spearman’s rank correlation was used to examine the association between MY-MINDD© scores and brain activation. Raw MY-MINDD© scores are displayed on the *x*-axis for interpretability; ranking was applied only in the correlation analysis. (**a**) Activated brain region when performing the 0-back task at the group level (*p* < 0.05, FWE-corrected). (**b**) Activated brain region when performing the 1-back at the group level (*p* < 0.05, FWE-corrected). (**c**,**d**) Scatterplots of positive association between MY-MINDD© scores and DLPFC activation. FWE, familywise error; DLPFC, dorsolateral prefrontal cortex; MFG, middle frontal gyrus.

**Figure 5:**
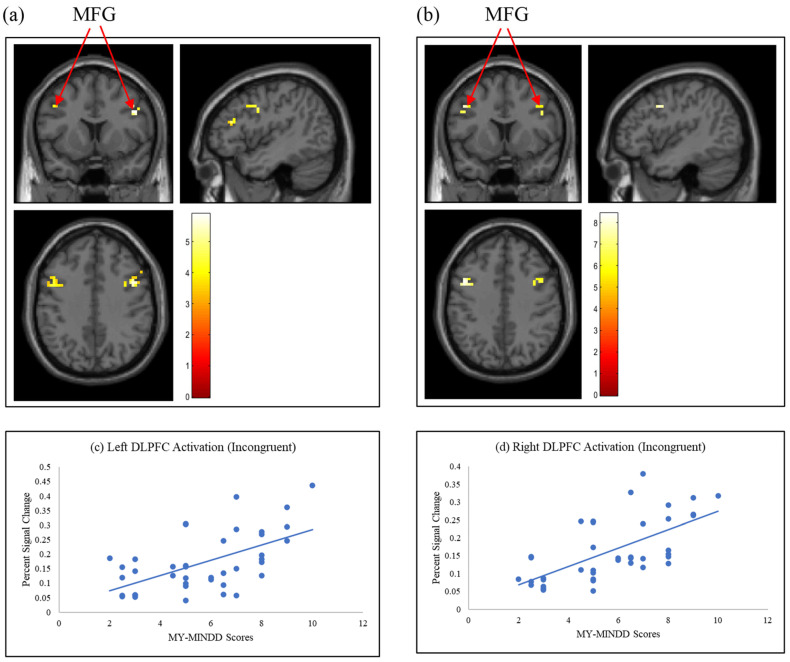
Spearman’s rank correlation was used to examine the association between MY-MINDD© scores and brain activation. Raw MY-MINDD© scores are displayed on the *x*-axis for interpretability; ranking was applied only in the correlation analysis. (**a**) Activated brain region when performing the SCWT-Congruent at the group level (*p* < 0.05, FWE-corrected). (**b**) Activated brain region when performing the SCWT-Incongruent at the group level (*p* < 0.05, FWE-corrected). (**c**,**d**) Scatterplots of positive association between MY-MINDD© scores and DLPFC activation. FWE, familywise error; DLPFC, dorsolateral prefrontal cortex; MFG, middle frontal gyrus.

Table 6 and Figure 4 and Figure 5 show the activated brain regions during the N-back and SCWT tasks (*p* < 0.05, FWE-corrected). Note that the middle frontal gyrus showed the highest activation for both the N-back and SCWT conditions (*p* < 0.05, FWE-corrected). Meanwhile, the inferior frontal gyrus and precentral gyrus exhibited the other activated regions, with the total number of voxels activated for N-back and SCWT being 708 and 183 voxels, respectively.

## 4. Discussion

As the first study to examine the association between a culturally adapted MIND diet score in Malaysia with brain activation, our findings support the relevance of the MY-MINDD© scores as a dietary tool for assessing neurocognitive health among older adults. This study revealed that higher MY-MINDD© scores were associated with better task performance in 0-back, SCWT control, and SCWT congruent tasks and greater brain activation in both the right and left DLPFC during the N-back, SCWT control, and SCWT incongruent tasks. Although subjects in the higher MY-MINDD© quartiles had relatively higher BMI values, all analyses were adjusted for BMI, along with age, gender, and years of formal education, to minimise potential confounding effects on the observed associations. These findings are consistent with previous functional neuroimaging studies demonstrating that healthier dietary patterns are associated with increased activation in prefrontal regions, particularly the DLPFC, during tasks involving working memory and cognitive flexibility [9,14,22]. Findings from a previous study also established that higher MY-MINDD© scores were related to better cognitive outcomes, including global cognitive function (MMSE), attention and working memory (Digit Span), and visuo-spatial function (Visual Reproduction) [17]. These findings demonstrate the potential of MY-MINDD© to serve as a proxy for identifying the risk of cognitive outcomes.

The N-back task is widely used to examine working memory and cognitive control in older adults [31]. This task involves both passive maintenance and executive processing tasks, especially under increased task demands, making it particularly suitable for research on cognitive ageing [32]. A previous longitudinal study involving 569 community-dwelling older adults in Chicago, United States, reported that higher MIND diet scores were linked to improved cognitive performance, including working memory [25]. Similarly, a systematic review found that adherence to the MIND diet was positively associated with global cognition and specific domains, including reasoning, enhanced thinking, and memory [33].

The 11 food groups in the MY-MINDD©, comprising seven brain-healthy and four unhealthy groups, are rich in nutrients such as folate, vitamin E, flavonoids, and lutein-zeaxanthin, which have antioxidant and anti-inflammatory properties that could improve cognitive function [17]. These nutrients help protect brain cells by reducing oxidative stress and free-radical damage, which can contribute to cognitive decline as we age [11]. Omega-3 fatty acids, specifically docosahexaenoic acid (DHA), derived from deep-sea fish, play an important role in neuronal membranes by enhancing membrane fluidity. This may help reduce brain atrophy, which can lead to neuroinflammation and neurodegeneration, while modulating functional brain activity [18]. A cohort study in the United States and Canada found that, among healthy older adults, higher levels of omega-3 fatty acids are associated with improved memory and processing speed during the SCWT task [13].

The other specific nutrient in the MY-MINDD©, such as polyphenols, has been recognised as essential for preserving neuronal integrity and supporting processes such as neurogenesis and synaptic plasticity [34]. Polyphenols, on the other hand, contribute to brain health by activating antioxidant and anti-inflammatory pathways, which are essential for synaptic plasticity and brain activity [35]. A comprehensive review highlighted that dietary polyphenols may enhance brain activity by increasing levels of brain-derived neurotrophic factor (BDNF), thereby promoting the formation of new synapses and enhancing neural responsiveness [36]. These mechanisms provide a plausible biological basis for the observed associations between higher MY-MINDD© scores and greater brain activation in regions such as the DLPFC, which has also been reported in previous fMRI studies examining diet-related neural function [9,14,22].

This study also showed that higher MY-MINDD© scores were associated with improved cognitive processing strategies, adaptability, and flexibility, which are closely related to DLPFC activity. This is in line with previous fMRI studies reporting that diets rich in brain-supportive nutrients, as reflected in higher MY-MINDD© scores, are associated with increased prefrontal cortex activation and with the preservation of this region’s structural and functional integrity [14,22,23]. Findings from a randomised-controlled trial among older adults demonstrated an increase in right DLPFC activation when subjects performed the 1-back task and in left DLPFC activation during the performance of the SCWT congruent task following 12 weeks of *Cosmos caudatus* supplementation—a traditional Asian vegetable known to have the highest phenolic content in comparison to other common Malaysian traditional vegetables [9].

Regarding task performance data, subjects with higher MY-MINDD© adherence scores showed better task performance (correct response percentage) during the 0-back, SCWT control, and congruent tasks, rather than response time. This was consistent with the findings of a previous study, which showed that older subjects preferred to maintain accuracy over speed when completing the task [37]. In addition, a higher percentage of correct responses was associated with greater DLPFC activation during the 0-back and SCWT control tasks than during the other demanding tasks. This may indicate that subjects had limited time to fully engage this region, potentially affecting their ability to respond accurately during more demanding conditions. Previous neuroimaging studies have reported similar patterns, showing that increases in cognitive load or insufficient processing time were accompanied by declines in behavioural performance and brain activation [38].

Another key finding of this study was the highest DLPFC activation observed in the middle frontal gyrus during the fMRI scan, along with additional activation in the precentral gyrus and inferior frontal gyri. The prefrontal cortex, located in the frontal region of the brain, is capable of attention, executive function, and working memory [5]. Specifically, working memory has been associated with activity in the left superior frontal gyrus, the right middle frontal gyrus, and the right inferior frontal gyrus [39,40]. Previous studies have also demonstrated that the middle frontal gyrus is crucial for tasks such as word reading and numerical processing [9,40]. Given the significant right DLPFC activation during the N-back task, our results suggest that subjects with higher MY-MINDD© scores may exhibit greater right-hemispheric dominance during visual-spatial working memory tasks compared with individuals with lower scores [41].

In addition, previous research has highlighted the involvement of the middle and superior frontal gyri in the DLPFC during the SCWT task [9,14,29]. Consistent with these findings, the present study demonstrated that subjects with higher MY-MINDD© scores showed greater activation in both left and right DLPFC during the SCWT-incongruent task, with activity observed bilaterally, particularly during the word recognition task, which aligns with a previous study [42]. However, the prior literature has reported mixed results. Some studies showed stronger left hemisphere activation [9,14], while others suggested right-sided dominance [4,29,43]. These discrepancies could be attributed to differences in sample size, severity of cognitive decline, or the specific brain regions analysed [44]. This pattern of bilateral DLPFC engagement may reflect neural compensation, an adaptive mechanism in which older adults engage additional neural resources to preserve cognitive function in response to age-related changes [45,46]. During conflict-related tasks such as the SCWT, the ACC is crucial for detecting cognitive interference. Meanwhile, the prefrontal cortex is responsible for resolving it, emphasising the collaborative role of these regions in sustaining executive control with ageing [44].

The implications of these findings may differ across normal ageing and pathological ageing conditions. Changes in prefrontal cortex activation patterns during normal ageing may reflect compensatory neural mechanisms aimed at preserving cognitive performance despite age-related decline [47]. In contrast, vascular cognitive disorders and AD are associated with more pronounced structural and functional changes involving cerebrovascular dysfunction, neurodegeneration, and disrupted neural connectivity [48]. As the MIND diet was originally developed to target neurodegenerative pathways through anti-inflammatory and antioxidant mechanisms, greater adherence to MY-MINDD© may potentially support neural function not only during normal cognitive ageing but also among individuals at increased risk of vascular-related cognitive decline and AD [25,49]. However, further longitudinal and interventional studies are needed to determine whether these neurofunctional mechanisms differ across specific disease conditions using neuroimaging approaches.

### Limitations

This study has a few limitations. Firstly, the relatively small sample size, while comparable to many fMRI studies due to the resource-intensive nature of neuroimaging protocols, may restrict the generalizability of the findings to the wider population of older adults. In addition, recruitment of older adults for fMRI studies is often constrained by stricter eligibility criteria and MRI-related contraindications, further limiting sample size. While the calculated sample size provided sufficient power for analyses using continuous MY-MINDD© scores, dividing the sample into quartiles yielded relatively small subgroups (approximately 10 subjects each). Such small group sizes may reduce statistical power and increase variability in brain activation measures. Thus, results from quartile-based comparisons should be interpreted with caution. Secondly, since the study is a cross-sectional design, it cannot determine causal relationships between MY-MINDD© adherence and brain activation. It remains unclear whether diet directly influences neural changes over time or if individuals with better cognitive function are more likely to follow healthier dietary patterns. Third, dietary intake was evaluated through a self-reported FFQ, which relies on subjects’ memory and honesty. This method may introduce recall bias and under- or over-reporting of certain food groups, which will affect the total MY-MINDD© scores. This study also relied exclusively on task-based fMRI, and resting-state functional connectivity data were not collected. Resting-state measurements could provide complementary information regarding spontaneous neural network organisation and would allow a clearer distinction between baseline functional differences and stimulus-driven activity. Lastly, although the fMRI tasks used were well-validated, the focus on specific ROIs, such as DLPFC, may overlook the involvement of other brain regions associated with cognitive function.

## 5. Conclusions

To conclude, this study provides preliminary evidence that greater adherence to the MY-MINDD© diet was associated with better task performance and greater DLPFC activation, as measured by fMRI. These findings are consistent with previous neuroimaging studies suggesting that adherence to brain-healthy dietary patterns may support neural activation in regions involved in executive function and working memory. These findings suggest that diet quality, as assessed using MY-MINDD©, may serve as a proxy for identifying risk of cognitive outcomes. Importantly, the present study extends existing evidence by demonstrating these associations using a culturally adapted MIND diet score among Malaysian older adults. Future studies with larger samples, longitudinal follow-up, and more comprehensive neuroimaging, such as whole-brain analysis, resting-state, structural, and multimodal fMRI, are needed to further validate and expand current findings.

## Figures and Tables

**Table 1:** Sociodemographic and health characteristics of subjects according to MY-MINDD©’s quartiles.

Characteristics	All (*n* = 40)	MY-MINDD© Score (Range 0–11)				*p* Value
		Q1 (0–3) (*n* = 10)	Q2 (4–5) (*n* = 10)	Q3 (6–7) (*n* = 10)	Q4 (8–11) (*n* = 10)	
MY-MINDD© score ^2^	5.68 ± 2.21	2.70 ± 0.35	4.90 ± 0.21	6.60 ± 0.39	8.50 ± 0.71	<0.001
Gender ^3^						0.865
Male	11 (27.5)	2 (20.0)	2 (20.0)	4 (40.0)	3 (30.0)	
Female	29 (72.5)	8 (80.0)	8 (80.0)	6 (60.0)	7 (70.0)	
Age ^1^	67.48 ± 2.99	66.50 ± 3.06	67.30 ± 1.95	67.20 ± 2.94	68.90 ± 3.67	0.335
Ethnicity ^3^						0.705
Malay	18 (45.0)	3 (30.0)	4 (40.0)	6 (60.0)	5 (50.0)	
Chinese	18 (45.0)	6 (60.0)	4 (40.0)	3 (30.0)	5 (50.0)	
Indian	4 (10.0)	1 (10.0)	2 (20.0)	1 (10.0)	0 (0.0)	
Marital status ^3^						0.920
Single	2 (5.0)	1 (10.0)	1 (10.0)	0 (0.0)	0 (0.0)	
Married	31 (77.5)	7 (70.0)	7 (70.0)	8 (80.0)	9 (90.0)	
Widow/Widower	7 (17.5)	2 (20.0)	2 (20.0)	2 (20.0)	1 (10.0)	
Household income ^2^ (RM)	5062.50 ± 3117.75	4700.00 ± 2626.79	3650.00 ± 2848.49	5800.00 ± 3259.18	6100.00 ± 3510.30	0.381
Education level ^3^						0.357
Secondary school	22 (55.0)	4 (40.0)	7 (70.0)	6 (60.0)	5 (50.0)	
Diploma	7 (17.5)	3 (30.0)	2 (20.0)	2 (20.0)	0 (0.0)	
Degree	11 (27.5)	3 (30.0)	1 (10.0)	2 (20.0)	5 (50.0)	
Formal education ^2^ (years)	13.63 ± 1.85	14.10 ± 1.85	13.00 ± 1.63	13.40 ± 1.84	14.00 ± 2.11	0.447
Body mass index ^1^ (kg/m^2^)	24.68 ± 3.40	23.77 ± 2.78	24.85 ± 2.14	22.96 ± 2.48 ^a^	27.13 ± 4.55 ^a^	0.030
Hypertension ^3^						0.689
Yes	26 (65.0)	7 (70.0)	6 (60.0)	5 (50.0)	8 (80.0)	
No	14 (35.0)	3 (30.0)	4 (40.0)	5 (50.0)	2 (20.0)	
Diabetes ^3^						0.853
Yes	6 (15.0)	1 (10.0)	2 (20.0)	2 (20.0)	1 (10.0)	
No	34 (85.0)	9 (90.0)	8 (80.0)	8 (80.0)	9 (90.0)	
Hyperlipidemia ^3^						0.627
Yes	24 (60.0)	7 (70.0)	6 (60.0)	7 (70.0)	4 (40.0)	
No	16 (40.0)	3 (30.0)	4 (40.0)	3 (30.0)	6 (60.0)	
Smoking ^3^						N/A
Yes	0 (0.0)	0 (0.0)	0 (0.0)	0 (0.0)	0 (0.0)	
No	40 (100.0)	10 (100.0)	10 (100.0)	10 (100.0)	10 (100.0)	
Social activity ^3^						0.865
Frequent	17 (42.5)	4 (40.0)	4 (40.0)	4 (40.0)	5 (50.0)	
Sometimes	14 (35.0)	4 (40.0)	2 (20.0)	5 (50.0)	3 (30.0)	
Seldom	6 (15.0)	1 (10.0)	2 (20.0)	1 (10.0)	2 (20.0)	
None	3 (7.5)	1 (10.0)	2 (20.0)	0 (0.0)	0 (0.0)	
Exercise ^3^						0.669
Everyday	11 (27.5)	3 (30.0)	2 (20.0)	1 (10.0)	5 (50.0)	
3–5 times per week	10 (25.0)	3 (30.0)	3 (30.0)	2 (20.0)	2 (20.0)	
1–2 times per week	15 (37.5)	3 (30.0)	3 (30.0)	6 (60.0)	3 (30.0)	
None	4 (10.0)	1 (10.0)	2 (20.0)	1 (10.0)	0 (0.0)	
GDS ^3^						0.551
No depressive symptoms	38 (95.0)	10 (100.0)	10 (100.0)	9 (90.0)	9 (90.0)	
Depressive symptoms	2 (5.0)	0 (0.0)	0 (0.0)	1 (10.0)	1 (10.0)	
IADL ^2^	7.78 ± 0.48	7.70 ± 0.68	7.90 ± 0.32	7.70 ± 0.48	7.80 ± 0.42	0.760

MY-MINDD©: Malaysian version of the MIND. GDS: Geriatric Depression Scale. IADL: Instrumental Activities of Daily Living. ^1^ One-way ANOVA test. ^2^ Kruskal–Wallis’s test. ^3^ Fisher’s Exact test was used to compare distributions across the quartiles of diet score. ^a^ Significant at *p* < 0.05 using Tukey’s Honestly Significant Difference (HSD) post hoc test. N/A = not applicable; no statistical comparison was performed because all participants were non-smokers.

**Table 2:** Univariate Analysis of Covariance (ANCOVA) Between MY-MINDD© Scores and Brain Activation.

Brain Activation	MY-MINDD© Score (Range 0–11)				*p* Value	Effect Size Cohen’s f
	Q1 (0–3) (*n* = 10)	Q2 (4–5) (*n* = 10)	Q3 (6–7) (*n* = 10)	Q4 (8–11) (*n* = 10)		
0-back left DLPFC activation	0.06 ± 0.08 ^a^	0.11 ± 0.11 ^b^	0.08 ± 0.07 ^c^	0.21 ± 0.13 ^abc^	0.026 *	0.25
0-back right DLPFC activation	0.11 ± 0.11 ^a^	0.15 ± 0.07	0.19 ± 0.05	0.23 ± 0.14 ^a^	0.044 *	0.22
1-back left DLPFC activation	0.05 ± 0.10 ^a^	0.16 ± 0.15	0.15 ± 0.12	0.24 ± 0.21 ^a^	0.104	0.17
1-back right DLPFC activation	0.10 ± 0.12 ^ab^	0.18 ± 0.16	0.25 ± 0.13 ^a^	0.26 ± 0.17 ^b^	0.043 *	0.22
SCWT Control Left DLPFC activation	0.02 ± 0.03 ^a^	0.19 ± 0.31 ^b^	0.19 ± 0.21	0.12 ± 0.10	0.211	0.13
SCWT Congruent Left DLPFC activation	0.07 ± 0.08	0.25 ± 0.36	0.13 ± 0.20	0.18 ± 0.14	0.376	0.09
SCWT Incongruent Left DLPFC activation	0.11 ± 0.06 ^d^	0.16 ± 0.09 ^e^	0.16 ± 0.14 ^f^	0.26 ± 0.09 ^def^	0.008 *	0.31
SCWT Control Right DLPFC activation	0.02 ± 0.03 ^a^	0.21 ± 0.35 ^a^	0.17 ± 0.20	0.12 ± 0.13	0.176	0.14
SCWT Congruent Right DLPFC activation	0.07 ± 0.07	0.21 ± 0.30	0.12 ± 0.16	0.17 ± 0.12	0.429	0.08
SCWT Incongruent Right DLPFC activation	0.09 ± 0.03 ^de^	0.14 ± 0.08 ^f^	0.19 ± 0.09 ^d^	0.22 ± 0.08 ^ef^	0.002 *	0.36

^a,b,c^ Significant at *p* < 0.05 using the least significance difference (LSD) post hoc test. ^d,e,f^ Significant at *p* < 0.01 using least significance difference (LSD) post hoc test. The model was adjusted for age, gender, years of education, and BMI. DLPFC, dorsolateral prefrontal cortex; SCWT, Stroop Colour Word Test. * Significant at *p* < 0.05.

**Table 3:** Relationship Between MY-MINDD© Scores and Task Performance.

Task Performance	MY-MINDD© Scores (Range 0–11)	
	Correlation (*ρ*)	*p*-Value
0-back correct response %	0.536 *	<0.001
1-back correct response %	0.041	0.802
0-back response time	−0.296	0.064
1-back response time	−0.112	0.492
SCWT control correct response %	0.737 *	<0.001
SCWT congruent correct response %	0.616 *	<0.001
SCWT incongruent correct response %	0.069	0.671
SCWT control response time	−0.301	0.059
SCWT congruent response time	−0.276	0.084
SCWT incongruent response time	−0.034	0.836

* Significant at *p* < 0.001. MY-MINDD©: Malaysian version of the MIND. SCWT: Stroop Colour Word Test.

**Table 4:** Relationship Between Task Performance and Brain Activation.

Task Performance	Correlation, *ρ* (*p*-Value)									
	0-Back DLPFC Activation		1-Back DLPFC Activation		SCWT Control DLPFC Activation		SCWT Congruent DLPFC Activation		SCWT Incongruent DLPFC Activation	
	L	R	L	R	L	R	L	R	L	R
0-back correct response %	0.523 (0.001) **	0.425 (0.006) **								
0-back response time	−0.335 (0.035) *	−0.184 (0.257)								
1-back correct response %			0.002 (0.990)	0.055 (0.738)						
1-back response time			−0.041 (0.802)	−0.086 (0.600)						
SCWT control correct response %					0.326 (0.040) *	0.404 (0.010) *				
SCWT control response time					−0.093 (0.569)	−0.010 (0.949)				
SCWT congruent correct response %							−0.045 (0.781)	−0.014 (0.931)		
SCWT congruent response time							−0.196 (0.226)	−0.048 (0.770)		
SCWT incongruent correct response %									−0.178 (0.272)	−0.078 (0.633)
SCWT incongruent response time									−0.112 (0.489)	0.015 (0.925)

* Significant at *p* < 0.05. ** Significant at *p* < 0.01. MY-MINDD©: Malaysian version of the MIND. DLPFC: dorsolateral prefrontal cortex. SCWT: Stroop Colour Word Test. L: left; R: right.

**Table 5:** Association Between MY-MINDD© Scores with Brain Activation.

Variables	Beta Coefficient, β (95% CI)							
	0-Back DLPFC Activation		1-Back DLPFC Activation		SCWT Control DLPFC Activation		SCWT Incongruent DLPFC Activation	
	L	R	L	R	L	R	L	R
**Unadjusted Model**								
**MY-MINDD© score**	0.478 (0.010–0.039) *	0.529 (0.012–0.038) *	0.421 (0.009–0.053)	0.444 (0.010–0.051) *	0.212 (−0.010–0.048)	0.176 (−0.014–0.048)	0.534 (0.013–0.041) *	0.629 (0.015–0.035) *
Adjusted Model **								
**MY-MINDD© score**	0.461 (0.008–0.039) *	0.564 (0.012–0.041) *	0.418 (0.008–0.054)	0.480 (0.012–0.054) *	0.198 (−0.013–0.049)	0.166 (−0.017–0.049)	0.567 (0.013–0.044) *	0.654 (0.015–0.036) *

* Significant at *p* < 0.0125. ** The model was adjusted for age, gender, years of education, and BMI. MY-MINDD©: Malaysian version of the MIND. CI: confidence interval. DLPFC: dorsolateral prefrontal cortex. SCWT: Stroop Colour Word Test. L: left; R: right.

**Table 6:** Activated Brain Regions When Performing N-back and SCWT task (*p* < 0.05, FWE-corrected).

Anatomical Region	L/R	Coordinates x, y, z	Voxels Activated	Maximum T Value
**0-back**				
Middle frontal gyrus	L	−54 17 32	84	8.40
Inferior frontal gyrus	R	45 8 38	147	8.10
**1-back**				
Middle frontal gyrus	L	−51 29 35	147	11.03
Middle frontal gyrus	R	39 41 35	330	11.10
**SCWT-Control**				
Middle frontal gyrus	L	−42 5 38	6	5.56
Middle frontal gyrus	R	51 8 38	14	5.59
**SCWT-Congruent**				
Precentral gyrus	L	−36 47 32	54	7.89
Middle frontal gyrus	R	36 50 32	66	8.25
**SCWT-Incongruent**				
Middle frontal gyrus	L	−57 20 32	25	7.45
Inferior frontal gyrus	R	60 23 26	18	6.25

L: left; R: right. SCWT: Stroop Colour Word Test.

## Data Availability

The datasets generated and/or analysed during the current study are available from the corresponding author on request, subject to approval by the relevant ethics committee and institutional guidelines.

## Abbreviations

The following abbreviations are used in this manuscript:

MY-MINDD©	Malaysian-MIND Diet
DLPFC	Dorsolateral Prefrontal Cortex
DASH	Dietary Approaches to Stop Hypertension
ACC	Anterior Cingulate Cortex
ROI	Region of Interest
SCWT	Stroop Colour Word Test
GLM	General Linear Model
fMRI	Functional Magnetic Resonance Imaging
SPM	Statistical Parametric Mapping
MNI	Montreal Neurological Institute
RFX	Random Effect Analysis
AAL	Automated Anatomical Labelling
FWE	Familywise Error
ANCOVA	Analysis of Covariance
MCI	Mild Cognitive Impairment
RAVLT	Rey Auditory Verbal Learning Test
IADL	Instrumental Activities of Daily Living
BMI	Body Mass Index
MMSE	Mini-Mental State Examination
FFQ	Food Frequency Questionnaire
GDS	Geriatric Depression Scale
CI	Confidence Interval
MFG	Middle Frontal Gyrus
DHA	Docosahexaenoic Acid
